# An investigation of the patterns and outcomes of Electroencephalographic (EEG) recording requests in the management of neuropsychiatric disorders in a teaching Hospital in Nigeria

**DOI:** 10.4314/ahs.v17i3.28

**Published:** 2017-09

**Authors:** Peter Omoniyi Ajiboye, Olatunji Alao Abiodun, Alexander Ikponmwosa Ogbebor

**Affiliations:** 1 Department of Behavioural Sciences University of Ilorin/ University of Ilorin Teaching Hospital; 2 Department of Behavioural Sciences University of Ilorin Teaching Hospital

**Keywords:** Electroencephalography, investigations, neuropsychiatric, Nigerian, Teaching Hospital

## Abstract

**Objective:**

To evaluate the relevance of Electroencephalography (EEG) in the management of various neuropsychiatric conditions in University of Ilorin Teaching Hospital (UITH), Ilorin.

**Background:**

EEG is still relevant in the diagnosis and management of patients with seizure disorders and extends to other neuropsychiatric conditions. However, very few studies have examined the use of EEG in developing countries, including Nigeria.

**Methods:**

The EEG records of 154 patients between January 2012 and December 2012 were reviewed. EEG unit's records, including EEG request forms and EEG reports were examined. Socio demographic data, clinical data and the neurologist's comments on the EEG recordings were extracted and recorded on the proforma form.

**Results:**

A total of 142(92.2%) of the patients out of 154 had complete records and were studied. Majority (84.5%) of the patients were below the age of 30 years. Various types of seizure disorders accounted for 80% of the provisional diagnosis. The EEG diagnosis based on the interpretation of the EEG records showed that 96 (67.6%) of the patients had normal records.

**Conclusion:**

EEG still plays a very important role in the investigation of neuropsychiatric conditions especially epilepsy in developing countries. EEG facilities should be readily available.

## Introduction

Electroencephalography (EEG) which was discovered by Hans Berger as far back 1929, is the recording of the electrical activities of the brain^1^. It is a surface recording of evoked potentials from brain neurons, referred to as evoked “field potentials”. It is an easily accessible, noninvasive test of brain neuronal functions, and provides information that primarily concerns disturbances of function, rather than structure^2^. EEG is still relevant in the diagnosis and management of patients with seizure disorders but its main clinical application, is for classification 1–5. Its diagnostic relevance also extends to conditions such as cerebrovascular diseases, head injuries, psychiatric illnesses and encephalopathy^3^,^4^,^5^. Other areas where EEG has clinical applications are states of altered consciousness including post-anoxic and traumatic coma, the parasomnias, dementia, toxic confusional states, cerebral infections and various other encephalopathies^2^.

In clinical psychiatry, EEG is also used in evaluating presence of seizure especially the ones that produce complex behaviors (temporal lobe, frontal lobe and petit mal seizures). EEG can also be used during electroconvulsive therapy (ECT) to monitor whether or not the stimulus produces seizure activity. In addition, EEG is a key component of polysomnography used in the evaluation of sleep disorders^6^.

As a result of a variety of conditions in which EEG is used as a diagnostic tool, some studies have questioned the appropriateness or otherwise of its use^7^,^8^. Even though some Nigerian authors reported that EEG services are limited and that the available ones are probably under- utilized^9^,^10^, authors from industrialized nations have reported over use or misuse of EEG as diagnostic and management tool^7^,^8^,^11^,^12^.

However, EEG is still of relevance in the investigation of neuropsychiatric conditions presenting to the Consultation-liaison (C-L) psychiatric settings especially indeveloping countries where financial constrains could be a major challenge when it comes to carrying out some neuroimaging procedures on patients. At our Centre, most requests for EEG come from the non-psychiatric departments of the hospital, served by the C-L psychiatric unit. The aim of the present study was to carry out retrospective review of all requests for EEG sent to the EEG unit of the hospital, and evaluate its relevance in the management of various neuropsychiatric conditions.

## Methods

### Study setting

The study was carried out at EEG unit of Department of Behavioural Sciences (which is the clinical Department of Psychiatry), University of Ilorin Teaching Hospital (UITH), Ilorin. UITH, Ilorin is a tertiary health institution owned by the Federal Government. The hospital is located in Ilorin, an urban Centre and capital of Kwara state of Nigeria. It is the only tertiary health facility in the state, with a primary catchment area of 2.3 million people^13^. Referrals to the hospital also come from neighbouring states. Kwara state is located in the North-Central zone of Nigeria.

### Subjects

This consisted of all patients referred to the EEG unit between January 2012 and December 2012. The choice of 2012 was based on the fact that most of the reports on the EEG recordings of patients, in the previous 5 years before the year 2012 were not available (Table 1).

**Table 1:** Patients registered for EEG and those with available reports

Year	Number of patients registered for EEG	Number of patients with EEG report (%)
2007	160	29 (18.1)
2008	180	57 (31.7)
2009	110	30 (27.2)
2010	131	19 (14.5)
2011	130	44 (33.8)
2012	154	142 (92.2)

### The EEG Unit

The EEG unit was established in the hospital in 1982, and domiciled in the Department of Behavioural Sciences (the clinical Department of Psychiatry) of the hospital. The EEG unit is managed by a Chief Electro-physiologist, assisted by two Technologists. The Chief Electro-physiologist and the two Technologists recorded the EEG of all the patients who were referred to the unit. When indicated, activation procedures such as hyperventilation and photic stimulation were also done. Interpretation of the EEG traces was done by a consultant Neurologist from the Department of Medicine of the same hospital.

### Procedure for the study

All patients who were referred for EEG between January 2012 and December 2012 were identified by examining EEG unit's records including the EEG request forms and EEG reports. The authors used a specially designed proforma form to record socio-demographic data such as the age, gender, and clinical data such as source of referral, provisional diagnosis by referring doctors, whether or not the patient was subjected to activation procedure (such as hyperventilation, photic stimulation or sleep recording). EEG abnormalities documented were recorded. The Neurologist's comments on each EEG recording were also extracted and recorded on the proforma form. We went further to explore whether or not EEG findings confirmed provisional diagnosis and whether or not EEG findings led to change of provisional diagnosis. Ethical approval to conduct the study was obtained from the hospital's ethics committee.

### Data analysis

Data analysis was done using Statistical Package for Social Sciences (SPSS) software, version 20. Simple frequency tables were generated, and used to present the data. Qualitative variables were summarized using frequencies and percentages. Quantitative variables were summarized using means, standard deviation and range. Chi- Square test was used to test for significant differences and P value less than 0.05 was set as the level for significant difference. Multiple regression analysis was done to see if there were any associations of abnormal EEG with socio-demographic characteristics and seizure types. The Coefficient of Determination (R2), which is the percentage of the variation in the outcome that can be explained by the model, was also computed.

## Results

### Sociodemographic characteristic of patients

A total of 142(92.2%) patients out of the 154 referred for EEG had complete results that could be traced. Majority 89 (62.7%) of them were males, mean age was 17.1 years, and range was 17.1 ± 14.1 years.

The age distributions of the patients were as follows: Majority, 59 (41.5%) were in the age bracket (0–10 years), 35 (24.7%) were between 11–20 years and 26 (18.3%) were between 21–30 years (Table 2).

**Table 2:** Socio-demographic characteristics of patients referred for EEG

Variables	Number of patients	Percentage
Gender		
Male	89	62.7
Female	53	37.3
Age		
0–10	59	41.5
11–20	35	24.7
21–30	26	18.3
31–40	12	8.5
41–50	4	2.8
51–60	6	4.2
Total	142	100

### Source s of referral

Almost a third (30.3%) were from internal Medicine department, 40 (28.2%) and 35 (24.6%) were from family medicine, and pediatric departments respectively (Table 3)

**Table 3:** Sources of referral

Variables	Number of patients	Percentage
Medicine	43	30.3
Family Medicine	40	28.2
Paediatrics	35	24.6
Behavioural Sciences (psychiatry)	16	11.3
Others+	8	5.6
Total	142	100

Note: + referrals from other hospitals in town

### Provisional diagnosis by the referring doctors

Various types of seizure disorders accounted for 80% of the provisional diagnosis. Seizure disorders (non-specific) accounted for 48.6% of the patients; generalized tonic clonic seizure (9.9%), complex partial seizure (5.6%), absence seizure and focal seizure (each 2.1%), infantile spasm, febrile seizure and idiopathic seizure (each 2.8%), cerebral palsy (8.5%) (Table 4).

**Table 4:** Provisional diagnosis by the referring doctors

Diagnosis	Number of patients	Percentage
Generalized tonic	14	9.9
clonic seizure		
Complex partial	8	5.6
seizure		
Absence seizure (Petit	3	2.1
mal)		
Simple partial seizure	2	1.4
Complex generalized	1	0.7
seizure		
Seizure disorders	69	48.6
(Unclassified)		
Febrile seizure	4	2.8
Infantile spasm	4	2.8
Focal seizure	3	2.1
Idiopathic seizure	4	2.8
Cerebral palsy	12	8.5
ADHD + Generalized	1	0.7
seizure		
Atonic seizure	1	0.7
Others⌽	12	8.5
Total	142	100

⌽ Others included extrapyramidal reaction (1), schizophrenia (1), post cerebral malaria neurological squeal (4), microcephaly (1), hyperkinetic disorder (1), migraine (1), no seizure (1), diagnosis not stated (2)

### EEG findings

The EEG diagnosis based on the interpretation of the EEG records by the neurologists showed that 96 (67.6%) of the patients had normal records while 46 (32.4%) had abnormal records. The breakdown of the abnormal records showed that generalized seizure accounted for 43.5% while focal seizure accounted for 34.8% of cases. EEG findings resulted in change of diagnosis in 8(5.6%) patients (Table 5).

**Table 5:** EEG Procedures and findings

Clinical activity	Patients N=142	Percentage
**Activation Procedure:**		
Yes	46	32.4
No	96	67.6
**EEG Findings led to change in diagnosis:**		
Yes	8	5.6
No	134	94.4
**EEG findings confirmed provisional diagnosis:**		
Yes	53	37.3
No	89	62.7
**EEG Diagnosis :**		
Normal EEG record	96	67.6
Abnormal EEG record	46	32.4
Specific diagnosis of the abnormal EEG record (N=46):		
Generalized seizure disorder	20	43.5
Focal seizure	16	34.8
Seizure disorder (nonspecific)	5	10.8
Benign Rolandic epilepsy	1	2.2
Infantile spasms (West syndrome)	1	2.2
Diffuse brain injury	3	6.5

### Regression analysis

Demographic factors found to be associated with having abnormal EEG included being older than 30 years of age (Odds ratio (OR) =0.529, Confidence interval (CI) =0.180–1.560, P = 0.249), and being a male (OR = 1.376, CI = 0.661– 2.865, P = 0.394) but the associations were not statistically significant. These two variables (age and male gender) were also not the best predictors of having abnormal EEG (R2 = 0.019; Predictive value = 67.6%; X2 = 1.895; P value = 0.388 (Table 6).

**Table 6:** Predictors of abnormal EEG

Variables	Odds ratio	95% CI for OR	*P* value
Age(> 30)	0.529	0.180 – 1.560	0.249
Sex (Male)	1.376	0.661 – 2.865	0.394

OR: Odds ratio; 95% CI: 95% Confidence Interval

## Discussion

The results of this study show that more males 89 (62.7%) than females 53 (37.3%) were referred for EEG and 84.5% of the patients were less than 30 years old. Preponderance of male referrals over females for EEG reported in this study is similar to reports from two previous Nigerian studies and studies from USA^9^–^11^, but differs from that of Pearce and Cock, from United Kingdom, which reported a preponderance of females over male referrals^8^. It has been reported that male preponderance may be a reflection of the fact that more male patients than females are affected by epilepsy. Epilepsy has been found to be the most common clinical condition among patients referred for EEG^9^,^10^,^14^,^15^.

Majority (84.5%) of the patients referred for EEG in this study were below the age of 30 years. This finding is similar to findings from other studies which have reported that majority of patients referred for EEG were below 30 years^9^,^10^,^16^,^17^. It however differs from that of a study which reported majority of their patients to be aged 0 – 10 years^14^. This finding would suggest that patients who utilize EEG services are young and still in their productive ages. In order to adequately meet the health care needs of this population, efforts should be made to ensure that EEG services are available in most hospitals in developing countries.

Results of our study also showed that almost a third (30.3%) of the referrals to the EEG unit came from the internal medicine department followed by family medicine (28.2%), paediatric (24.6%) and behavioural sciences (psychiatry) (11.3%). The results of this study differ from that of Olisah et al, which reported highest referral ratefrom the general outpatient clinic (family medicine) [52%], followed by the department of psychiatry (28%)^9^. Our study would suggest that more departments are aware of, and make use of the EEG facility in investigating their patients. Some authors have however suggested that routine EEG examination has limited value as screening test for organic brain disorders^18^,^2^ and that an abnormal EEG is only helpful when it supports the suspicion of an organic disorder that is arrived at from findings on history and clinical evaluation of the patients^18^,^2^. As shown by the present study, it appears that use of EEG by psychiatrists (11.3% in present study) in tertiary Centres where there are neurologists, has declined since the neurology unit could handle epilepsy cases without associated complex behavioural problems. It is hoped that in future when more facilities become available, psychiatrists in developing countries will be able to extend electrical studies of the brain to sleep and sleep related disorders and other neuropsychiatric conditions^2^,^19^,^20^,^21^.

The provisional diagnosis of the referring doctors in this study showed that epilepsy (79.5%) was the commonest reason for referral, (with 30.9% having specific or classified diagnosis and 48.6% unclassified diagnosis), cerebral palsy 8.5% and some other minor conditions. The finding from this study that epilepsy was the commonest reason for which patients were referred for EEG is similar to some other studies in Nigeria^9^,^10^, Africa^14^ and Europe^12^ but differs from result of a study from USA^11^. Harmon et al, reported that the commonest reason for EEG referral was altered mental status 52 (26%) followed by seizure 48 (24%)^8^. Some other authors are of the opinion that the reason for requesting for EEG could vary between clinicians^7^. Smith et al, in their study reported that epilepsy and seizure accounted for (62%) of the referral from neurologists while other doctors used EEG procedure as a diagnostic tool as it was not uncommon for them to refer patients with clinical history of “funny turning” or “aggressive outburst,” to exclude epilepsy^7^.

The results of this study also showed that the EEG result was normal in 67.6% of the patients. This finding differs from previous reports that reported lower percentage of their patients with normal EEG reports^10^,^16^. However, a normal EEG does not exclude epilepsy. It is important to note that in many of the patients who had normal records it was a common occurrence to find EEG reports such as: “A normal record, this does not rule out seizure disorders.” Smith^1^, reported that around 10% of patients with epilepsy never show epileptiform discharge and that an abnormal EEG demonstrating interictal epileptiform discharge (IED) does not itself confirm that an individual has seizure disorder. This is because IED may be found in a small percentage of normal subjects who never develop epilepsy and IED could also be a finding in patients with other neurological disorders which are not complicated by epilepsy^1^. It implies that an abnormal EEG should always be considered in the context of the clinical assessment as well as other investigations. It should be used as supportive rather than conclusive evidence towards a diagnosis^12^.

EEG has low sensitivity in the diagnosis of epilepsy but better specificity. This is important if we also consider the fact that in this study our results show that EEG reportsconfirm provisional diagnosis in 37.3% of the patients and led to change of provisional diagnosis only in 5.6% of the patients. Some authors are of the opinion that substantial numbers of EEG requests are made inappropriately based on the misconception that EEG can confirm or exclude a diagnosis of epilepsy in patients with funny turns, black out and low clinical suspicion of epilepsy^7^, ^8^,^12^. There is therefore the need for clinicians to be educated on the fact that interictal EEG has its limitations and pitfalls and they need to be more selective in making requests for EEG.

Multiple regression analysis of associations of abnormal EEG (i.e. Various seizure types) with socio-demographic characteristics (gender and age) showed that gender and age were not predictors of abnormal EEG in the study population. This will be an area of focus in future studies because of its clinical and management importance in clinical practice.

## Conclusion

EEG still plays a very important role in the investigation of neuropsychiatric conditions especially epilepsy in developing countries. Efforts should therefore be made to ensure that hospitals in developing countries are equipped with EEG facilities. It is hoped that in future, psychiatrists from developing countries will be able to extend electrical studies of the brain to sleep and sleep related disorders and other neuropsychiatric conditions.

## Coflict of interest

There is no conflict of interest
